# A Standardized Onion Peel-Derived Bioactive Ingredient Attenuates Palmitate-Induced Steatosis and Oxidative Stress by Modulating Mitochondrial Dynamics and Autophagy in HepG2 Cells

**DOI:** 10.3390/antiox15040513

**Published:** 2026-04-21

**Authors:** Ilaria Di Gregorio, Vincenzo Migliaccio, Maria D’Elia, Rita Celano, Valentina Santoro, Anna Lisa Piccinelli, Mariateresa Russo, Luca Rastrelli, Lillà Lionetti

**Affiliations:** 1 Department of Chemistry and Biology “A. Zambelli”, University of Salerno, Via Giovanni Paolo II, 132, 84084 Salerno, Italy; idigregorio@unisa.it (I.D.G.); vmigliaccio@unisa.it (V.M.); 2 Department of Pharmacy, University of Salerno, Via Giovanni Paolo II, 132, 84084 Salerno, Italy; mdelia@unisa.it (M.D.); rcelano@unisa.it (R.C.); apiccinelli@unisa.it (A.L.P.); 3 National Biodiversity Future Center (NBFC), 90133 Palermo, Italy; 4 Dipartimento di Scienze della Terra e del Mare, University of Palermo, 90123 Palermo, Italy; 5 Department of Agriculture Science, Food Chemistry, Safety and Sensoromic Laboratory (FoCuSS Lab), University of Reggio Calabria, Via Salita Melissari, 89124 Reggio Calabria, Italy; mariateresa.russo@unirc.it

**Keywords:** onion peel bioactive ingredient, oxidative stress, mitochondrial dynamics, autophagy, palmitate-induced steatosis, HepG2 cells

## Abstract

Onion peel represents a valuable food by-product rich in bioactive phenolic compounds. Building on previous phytochemical investigations, an onion peel extract from the *Rossa* *di Tropea* variety was developed as a standardized bioactive ingredient (OPI-T), defined by flavonol (quercetin and its glycosylated and oxidized derivatives) and anthocyanin (cyanidin derivatives) markers, ensuring batch-to-batch consistency, and evaluated for its potential against hepatic steatosis. The present study aimed to assess the protective effects of OPI-T against palmitate-induced steatosis and oxidative stress in HepG2 cells, a widely used in vitro model of hepatic lipid accumulation. An onion peel extract derived from the *Ramata di Montoro* variety was included as a natural negative reference to account for varietal variability. HepG2 cells were co-treated with palmitate (500 µM) and OPI-T (25 or 50 µg/mL). Lipid accumulation was evaluated by Oil Red O and BODIPY staining, while oxidative stress was assessed by the DCF assay. Mitochondrial dynamics and autophagy were investigated through the analysis of key protein markers, including MFN2, DRP1, SQSTM1/p62 and LC3 II/I. OPI-T significantly attenuated palmitate-induced lipid accumulation (−18%) and reduced intracellular ROS production (−75%), while modulating mitochondrial dynamics toward a reduced fission phenotype with a marked increase in the MFN2/DRP1 ratio (1.66) and improving autophagy flux. In contrast, the *Ramata di Montoro* variety showed weaker or inconsistent effects under the same experimental conditions. Overall, these findings support the functional validation of a standardized onion peel-derived ingredient, highlighting its potential application as a bioactive component for functional food or nutraceutical development targeting hepatic steatosis and oxidative stress.

## 1. Introduction

Oxidative stress plays a key role in metabolic diseases associated with high-fat feeding and obesity, including insulin resistance and metabolic dysfunction-associated fatty liver disease (MASLD) [1]. MASLD encompasses a spectrum of liver alterations ranging from simple steatosis to metabolic dysfunction-associated steatohepatitis (MASH), characterized by inflammation and fibrosis [2]. Metabolic dysfunction underlies the close relationship between fatty liver disease and other obesity-related disorders such as type 2 diabetes and cardiovascular disease. Since pharmacological therapies for MASLD/MASH are still limited, dietary strategies and bioactive food components represent a primary intervention approach [3,4]. In this context, bioactive compounds derived from plant-based foods, particularly polyphenols, have attracted increasing interest due to their ability to modulate key pathological mechanisms involved in MASLD, including oxidative stress, inflammation, mitochondrial dysfunction, and metabolic dysregulation [5]. Several polyphenols, such as curcumin, resveratrol, and silymarin, have shown hepatoprotective effects in experimental models, supporting the investigation of plant-derived bioactive ingredients as potential tools for MASLD management [6].

Onion (*Allium cepa* L.) peel is a major by-product of onion processing and represents a rich source of bioactive phenolic compounds, particularly flavonols and anthocyanins. The increasing demand for processed onions has led to the accumulation of large amounts of peel waste, which is unsuitable for feed or landfill disposal due to its chemical composition and odor. Consequently, the valorization of onion peel as a source of bioactive ingredients fits well within current strategies aimed at sustainable food systems and circular economy approaches [7]. Celano et al. [8] provided a comprehensive phytochemical characterization of onion peel extracts obtained from two Italian Protected Geographical Indication (PGI) varieties, the red *Rossa di Tropea* and the coppery *Ramata di Montoro*. Using UHPLC-based approaches, the authors demonstrated that onion peel extracts are rich in flavonols, mainly quercetin and its derivatives, and anthocyanins, particularly cyanidin-3-glucoside. These extracts showed significant antioxidant and anti-diabetic activities in vitro, as well as the absence of cytotoxic effects on human dermal fibroblast (HDFA), highlighting their potential as bioactive ingredients for nutraceutical and functional applications [8].

While the antioxidant and metabolic effects of onion peel extracts have been established at the chemical and screening level, their functional validation in hepatic models relevant to MASLD remains limited. In particular, oxidative stress and lipid accumulation are tightly interconnected in hepatocytes, with mitochondrial dysfunction representing a central pathogenic event in steatosis development [9]. Therefore, the evaluation of onion peel-derived bioactive ingredients in cellular models of fatty acid-induced steatosis is a necessary step toward their translational application. Hepatic steatosis is characterized by excessive lipid accumulation in hepatocytes and can be experimentally induced in vitro by exposing liver cells to saturated fatty acids such as palmitate [10,11,12,13,14,15,16,17,18]. Palmitate overload leads to mitochondrial dysfunction, excessive reactive oxygen species (ROS) production, and alterations in mitochondrial dynamics, including increased fission and reduced fusion processes [19,20,21]. Dysregulated mitochondrial dynamics, involving proteins such as dynamin-related protein 1 (DRP1) and mitofusin 2 (MFN2), have been implicated in the progression of steatosis and steatohepatitis [22,23].

Autophagy plays a crucial role in hepatic lipid homeostasis by regulating lipid droplet degradation and mitochondrial quality control [24]. Although autophagy is initially activated during lipid overload, prolonged exposure to saturated fatty acids such as palmitate leads to autophagy impairment, contributing to steatosis progression [25,26]. Importantly, mitochondrial dynamics and autophagy are functionally interconnected, as mitochondrial fission facilitates mitophagy, while autophagy maintains mitochondrial network integrity [27]. In this context, the present study aimed to functionally validate a standardized onion peel-derived bioactive ingredient developed from the Rossa di Tropea variety in an in vitro model of palmitate-induced hepatic steatosis. HepG2 cells were used to evaluate lipid accumulation, oxidative stress, mitochondrial dynamics, and autophagy. An onion peel extract obtained from the *Ramata di Montoro* variety was included as a natural negative reference, allowing the assessment of whether the observed effects were specific to the standardized ingredient rather than reflecting a generic onion peel matrix effect.

## 2. Materials and Methods

### 2.1. Reagents and Materials

All reagents and solvents used in this study were of analytical grade. Sodium palmitate, fatty acid-free bovine serum albumin (BSA), Oil Red O, 3-(4,5-dimethyl-2-thiazolyl)-2,5-diphenyl-2H-tetrazolium bromide (MTT), 2′,7′-dichlorofluorescin diacetate (DCFH-DA), paraformaldehyde (PFA), Triton X-100, sodium deoxycholate, sodium dodecyl sulfate (SDS), and other general laboratory reagents were purchased from Sigma-Aldrich/Merck (Darmstadt, Germany), unless otherwise specified. Cell culture media and supplements, including Minimum Essential Medium (MEM), Dulbecco’s Modified Eagle Medium (DMEM), fetal bovine serum (FBS), non-essential amino acids, L-glutamine, penicillin, and streptomycin, were obtained from Euroclone S.p.A. (Milan, Italy). BODIPY™ 493/503 fluorescent probe was purchased from Invitrogen (Thermo Fisher Scientific, Waltham, MA, USA). Primary antibodies used for Western blot analysis included MFN2, DRP1, GAPDH (Santa Cruz Biotechnology, Dallas, TX, USA), P62/SQSTM1, LC3B (Cell Signaling Technology, Danvers, MA, USA) and alpha-Tubulin (ABCAM, Cambridge, CB2 0AX, UK). Unless otherwise stated, all aqueous solutions were prepared using ultrapure water.

### 2.2. Preparation and Standardization of the Onion Peel Ingredient (OPI-T)

Outer dry protective layers of brown skin onion bulbs (*Allium cepa* L.) of *Ramata di Montoro* were supplied by the company “Gaia Società Semplice Agricola” (Montoro, Avellino, Italy) located in the production area of onion PGI *Ramata di Montoro*. Outer dry protective layers of red skin onion bulbs (*Allium cepa* L.) of PGI *Rossa di Tropea* were provided from Calabrian farmers which organically cultivated and harvested *Rossa di Tropea* onions in Calabria region (Tropea, Vibo Valentia, Italy). Outer dry peels of *Allium cepa* L. cv. Rossa di Tropea (PGI) were collected after harvest, naturally dried, milled, and sieved to obtain a homogeneous powder, according to the procedure previously described by [8]. Particular attention was paid to raw material handling in order to ensure batch-to-batch reproducibility of the starting matrix. The onion peel ingredient from *Rossa di Tropea* (OPI-T) was obtained by exhaustive ultrasound-assisted solid–liquid extraction (UAE), selected as a scalable and mild technology suitable for ingredient development. Extraction was conducted using aqueous ethanol (70% *v*/*v*), as previously optimized [8]. This hydroalcoholic mixture was selected for its proven capacity to solubilize a wide range of phenolic compounds, including flavonols and anthocyanins, while ensuring food-grade safety, environmental sustainability, and reproducibility of the phytochemical profile. Briefly, dried onion peel powder was extracted using aqueous ethanol (70% *v*/*v*) with a matrix-to-solvent ratio of 1:20 (*w*/*v*). Extraction was performed in a thermostat-controlled ultrasound bath (20 kHz) at 25 °C for three consecutive cycles of 30 min each. After each cycle, the extract was centrifuged (10 min, 9000× *g*), and the supernatants were pooled. The combined extracts were filtered, concentrated under reduced pressure at 40 °C to remove ethanol, and subsequently freeze-dried to obtain a dry powder (OPI-T). The extraction yield was calculated as grams of dry extract per 100 g of dried onion peel. The dried ingredient was stored at −20 °C, protected from light and moisture, until further use. For comparison purposes, an onion peel extract obtained from *Allium cepa* L. cv. *Ramata di Montoro* (PGI) was prepared using the same extraction protocol but was not subjected to ingredient standardization (M). This extract was used as a natural reference control, allowing the evaluation of whether the biological effects observed were specifically associated with the standardized ingredient OPI-T rather than representing a general property of onion peel extracts.

### 2.3. Chemical Standardization and Quality Specifications of OPI-T

Chemical standardization of OPI-T was performed to define quality specifications suitable for its use as a bioactive ingredient, rather than to reproduce the exhaustive phytochemical characterization previously reported [8]. Based on the established chemical profile of *Rossa di Tropea* onion peel extracts, flavonols and anthocyanins were selected as the main markers of identity and functionality. Quantitative UHPLC–UV analysis was used to confirm the presence and relative abundance of quercetin and its main glycosylated and oxidized derivatives, along with cyanidin derivatives, which define the distinctive chemical profile of OPI-T.

Quercetin and cyanidin equivalents were adopted as primary marker compounds, and batch acceptance was defined by maintaining marker content within an acceptable variability range of ±10% across independently produced batches, based on internal analytical reproducibility criteria. Standardization of OPI-T was based on quantitative determination (Supplementary Materials) of polyphenolic markers defining its compositional identity. Total flavonols (quercetin and its glycosylated and oxidized derivatives) ranged from 103 to 126 mg g^−1^ (expressed as quercetin equivalents; RSD < 3%), while total anthocyanins (expressed as cyanidin-3-O-glucoside equivalents) ranged from 21 to 26 mg g^−1^ (RSD < 5%). These ranges establish the reference specifications for OPI-T quality and ensure consistency across production batches.

In addition, a minimum total flavonol content of 100 mg g^−1^ (expressed as quercetin equivalents) was defined as a secondary standardization criterion to ensure functional consistency of the ingredient. Only batches meeting these predefined quality specifications were used for biological assays. Ethanol removal was achieved under reduced pressure followed by freeze-drying, yielding OPI-T as a dry powder. The extraction and drying process was designed to ensure the absence of residual organic solvents, making the standardized ingredient suitable for biological testing and potential nutraceutical applications.

### 2.4. UHPLC–UV–HRMS/MS Analysis

The comparability of the chemical profiles between the extracts analyzed in this study and those previously investigated [8] was assessed using the UHPLC-HRMS^n^ and UHPLC-UV methods described in [8]. The purpose of this analysis was not an exhaustive recharacterization, but the verification of marker identity and chemical consistency of the onion peel ingredient used in the biological assays. A Vanquish Flex UHPLC system (Thermo Fisher Scientific, Milan, Italy) coupled to a diode array detector (PDA) (Thermo Fisher Scientific, Milan, Italy) and an Orbitrap Exploris 120 high-resolution mass spectrometer equipped with a heated electrospray ionization source (HESI-II) was used. Chromatographic separation was achieved on a Kinetex C18 column (2.1 × 50 mm, 1.7 µm; Phenomenex, Torrance, CA, USA), maintained at 30 °C. The mobile phase consisted of (A) H_2_O and (B) CH_3_CN, both containing 0.1% formic acid, at a flow rate of 600 µL/min. The elution gradient was programmed as follows: 0–3 min, 2% B; 3–5 min, 2–13% B; 5–9 min, 13% B; 9–12 min, 13–18% B; 12–13 min, 18% B; 13–17 min, 18–30% B; 17–21 min, 30–50% B; 21–22 min, 50–98% B; 22–27 min, 98% B. The injection volume was set at 5 µL. The UV spectra were acquired in the range of 200–600 nm and HRMS data were acquired in negative and positive ionization mode. MS data were acquired in Full MS/data-dependent MS^2^ (dd-MS^2^) mode, with resolving powers of 60,000 and 30,000 FWHM for Full MS and MS^2^ scans, respectively. Fragmentation was achieved using stepped higher-energy collisional dissociation (HCD) with collision energies set at 20, 40, and 60 eV. Phenolic compounds were identified by accurate mass measurement, diagnostic fragmentation, and retention time, in accordance with the characterization previously performed on the same extracts [8]. Flavonols, mainly quercetin and its glycosylated derivatives, were used as reference markers to confirm the chemical consistency of the standardized ingredient across batches. Instrument control and data processing were carried out using Xcalibur software version 4.6 (Thermo Fisher Scientific).

### 2.5. Experimental Design

Human hepatocellular carcinoma cells (HepG2) were obtained from the Interlab Cell Line Collection (Advanced Biotechnology Centre, Genoa, Italy). HepG2 cells are a widely used in vitro model for the study of hepatic metabolism, lipid accumulation, and oxidative stress-related mechanisms [28]. Cells were cultured in Minimum Essential Medium (MEM) supplemented with 10% (*v*/*v*) fetal bovine serum, 1% (*v*/*v*) non-essential amino acids, 0.2 mM L-glutamine, 50 U/mL penicillin, and 50 µg/mL streptomycin (Invitrogen Srl, Milan, Italy), and maintained at 37 °C in a humidified atmosphere containing 5% CO_2_. For experimental treatments, cells were seeded at a density of 5 × 10^4^ cells/cm^2^ and allowed to adhere overnight. Hepatic steatosis and oxidative stress were induced by treatment with palmitate (500 µM), conjugated with bovine serum albumin (BSA), as described above. Cells were simultaneously treated with the standardized onion peel ingredient obtained from *Allium cepa* L. cv. Rossa di Tropea (OPI-T) at concentrations of 6.25, 12.5, 25, and 50 µg/mL. The extract solutions were prepared by dissolving the powder in 70% ethanol, obtaining a stock concentration of 7 mg/mL. To ensure a homogeneous solution free from any residue, the batches of stock solution were vortexed for 1 min and centrifuged for 2 min at 13,300 rpm. Finally, the supernatant was collected and stored at −20 °C. At the time of treatment, appropriate dilutions were prepared in culture medium immediately prior to use. For comparison purposes, an onion peel extract obtained from *Allium cepa* L. cv. Ramata di Montoro (M) was included as a non-standardized natural reference control and tested at the same concentrations, dissolved in the same manner. In all experimental conditions, cells did not exceed 70% confluence at the time of treatment, avoiding the formation of multilayers typical of tumor cells such as HepG2. The treatment times used for HepG2 cells were 24 and 48 h, as these time intervals are widely used in in vitro models of hepatocellular carcinoma and allow for reliable assessment of cytotoxicity, gene expression, and metabolic effects, minimizing confounding factors associated with prolonged culture, such as nutrient depletion and overconfluence [29,30,31,32]. Furthermore, under our experimental conditions, 24 h of treatment of HepG2 cells with PA was sufficient to determine lipids and reactive oxygen species (ROS) accumulation and, therefore, to develop an in vitro model of hepatic steatosis, as also reported in the literature [33,34,35,36,37,38,39].

### 2.6. Preparation of Palmitate Solutions

Palmitate solutions were prepared as previously described, with minor modifications [40]. Briefly, sodium palmitate was dissolved in 0.1 M NaOH to obtain an 8 mM stock solution and heated at 70 °C for 15 min under constant stirring until complete solubilization. A 10% (*w*/*v*) fatty acid-free bovine serum albumin (BSA; Merck, Darmstadt, Germany) solution was prepared in 0.9% NaCl and maintained at 55 °C. The palmitate solution was then slowly added to the pre-warmed BSA solution to obtain a palmitate–BSA complex, which was incubated for at least 15 min in a shaking water bath at 55 °C to ensure complete binding. The palmitate–BSA stock solution was aliquoted and stored at −20 °C. At the time of treatment, the solution was diluted in cell culture medium to obtain a final palmitate concentration of 500 µM, as used to induce lipid accumulation and oxidative stress in HepG2 cells.

### 2.7. Cell Viability MTT Assay

Cell viability was assessed using a colorimetric MTT assay based on the mitochondrial enzymatic reduction of 3-(4,5-dimethyl-2-thiazolyl)-2,5-diphenyl-2H-tetrazolium bromide (MTT; M5655, Sigma-Aldrich, Darmstadt, Germany) to insoluble formazan crystals by metabolically active cells. The amount of formazan formed is proportional to the number of viable cells. HepG2 cells were seeded in 96-well plates and treated with BSA, palmitate, and the onion peel preparations as described above. At the end of the treatment period, MTT was added to each well at a final concentration of 0.5 mg/mL in 100 μL of culture medium and incubated for 90 min at 37 °C in a humidified atmosphere containing 5% CO_2_. After incubation, the culture medium was removed and the resulting formazan crystals were dissolved in dimethyl sulfoxide (DMSO). Absorbance was measured using a microplate (Multiskan GO, Thermo Fisher Scientific, Waltham, MA, USA) reader at 595 nm, with background subtraction at 655 nm. Cell viability was expressed as a percentage relative to control cells. Data were expressed as fold changes relative to the control group and derived from at least three independent experiments performed in triplicate.

### 2.8. Wound Healing (Scratch) Assay for Cell Migration Analysis

To further support cell viability and exclude cytotoxic effects of the treatments, a wound healing (scratch) assay was performed to evaluate cell migration. HepG2 cells were seeded in 24-well plates and grown to full confluence. After removal of the culture medium, a linear scratch was created across the cell monolayer using a sterile P10 pipette tip (Euroclone S.p.A. (Milan, Italy). Detached cells and debris were removed by washing with phosphate-buffered saline (PBS), and fresh culture medium was added. Images of the scratch area were acquired immediately after scratching (T0) using an inverted microscope (Olympus CKX41 Inverted Phase, Tokyo, Japan). Subsequently, cells were incubated with the complete treatment media and maintained at 37 °C. Images of the same scratch area were captured after 24 and 48 h. Cell migration was quantified by measuring the scratch width or area at each time point using Image Processing and Analysis in Java (ImageJ, version 1.54p released 18 February 2025, National Institutes of Health, Bethesda, MD, USA). Results were expressed as percentage wound closure relative to T0. Representative images and quantitative analyses were obtained from randomly selected fields in three independent experiments performed in triplicate.

### 2.9. Oil Red O Staining

Intracellular lipid accumulation was evaluated by Oil Red O staining followed by quantitative spectrophotometric analysis. HepG2 cells were treated with palmitate (500 µM) in combination with the onion peel preparations at two concentrations (25 and 50 µg/mL) for 24 and 48 h, as described above. The assay was performed according to a previously reported protocol, with minor modifications [41]. At the end of the treatment period, cells were washed with phosphate-buffered saline (PBS) and fixed with 4% paraformaldehyde (PFA) for 45 min at room temperature. After fixation, cells were washed twice with distilled water pre-heated to 37 °C and incubated with 60% isopropanol for 5 min. Oil Red O working solution was freshly prepared by filtering a saturated Oil Red O solution (Sigma-Aldrich, O0625) and diluting it in isopropanol:water (3:2, *v*/*v*). Cells were incubated with the staining solution for 10–20 min at room temperature. Excess dye was removed by washing with 100% isopropanol followed by distilled water.

For quantitative analysis, intracellular Oil Red O was eluted with 60% isopropanol, transferred to a 96-well plate, and absorbance was measured at 490 nm using a microplate reader. Lipid accumulation was expressed relative to non-stimulated control cells and normalized to protein concentration, determined using the Bradford assay. Data were expressed as fold changes relative to the control group and derived from at least three independent experiments performed in triplicate.

### 2.10. BODIPY Fluorescence Probe Staining for Microscopy

Intracellular lipid droplets were visualized using BODIPY 493/503 fluorescent staining. HepG2 cells (1 × 10^4^ cells/well) were seeded in 24-well plates and cultured overnight in Dulbecco’s Modified Eagle Medium (DMEM) at 37 °C in a humidified atmosphere containing 5% CO_2_. Cells were then treated with the appropriate experimental media for 24 h. At the end of the treatment period, cells were washed twice with phosphate-buffered saline (PBS) and fixed with 4% paraformaldehyde (PFA) for 20 min at room temperature. BODIPY staining solution (10 µM; Invitrogen™ BODIPY™ 493/503, D3922) was freshly prepared in PBS according to a previously described protocol [42] and added to the cells for 20 min at room temperature. After staining, cells were washed twice with PBS and lipid droplets were visualized using a fluorescence microscope equipped with a fluorescein isothiocyanate (FITC) filter at 20× magnification. Representative images analyses were obtained from randomly selected fields in three independent experiments performed in triplicate.

### 2.11. Intracellular ROS Measurement by DCFH-DA Assay

Intracellular reactive oxygen species (ROS) levels were determined using the 2′,7′-dichlorofluorescin diacetate (DCFH-DA) assay. HepG2 cells (1 × 10^6^ cells/well) were seeded in 24-well plates and cultured overnight in DMEM at 37 °C. Cells were then incubated with the experimental treatment media for 24 h. A 10 mM DCFH-DA stock solution was prepared by dissolving 4.85 mg of DCFH-DA (Sigma-Aldrich/Merck, D6883) in 1 mL of dimethyl sulfoxide (DMSO). Immediately before use, the stock solution was diluted in pre-warmed DMEM to obtain a 10 µM working solution. Cells were incubated with 500 µL of the DCFH-DA working solution at 37 °C for 30 min. After incubation, the dye-containing medium was removed, and cells were washed once with DMEM and twice with PBS. Cells were then lysed by adding 200 µL of radioimmunoprecipitation assay (RIPA) buffer per well and incubating on ice for 5 min. Cell lysates were collected, centrifuged at 21,130× *g* for 10 min at 4 °C, and 100 µL of the supernatants was transferred to a black 96-well plate. Fluorescence intensity was measured using a fluorescence microplate reader at an excitation wavelength of 485 nm and an emission wavelength of 530 nm. ROS levels were normalized to protein concentration, determined using the Bradford assay. Data were expressed as fold changes relative to the control group and derived from three independent experiments performed in triplicate.

### 2.12. Western Blotting Analysis

Western blot analysis was performed to evaluate the protein expression of markers involved in mitochondrial dynamics and autophagy. HepG2 cells were seeded in 6-well plates and treated with palmitate and onion peel preparations as described above. After 24 h, cells were lysed in 75 µL of RIPA buffer containing 150 mM NaCl, 1.0% Triton X-100, 0.5% sodium deoxycholate, 0.1% sodium dodecyl sulfate (SDS), and 50 mM Tris–HCl (pH 8.0), supplemented with a protease inhibitor cocktail (Aprotinin, Pepstatin, Leupeptin, sodium orthovanadate, and phenylmethylsulfonyl fluoride; Sigma-Aldrich). Cell lysates were collected using a cell scraper and centrifuged at 12,000× *g* for 15 min at 4 °C. Protein concentration was determined using the Bradford assay. Equal amounts of protein (30 µg) were separated by SDS–polyacrylamide gel electrophoresis (SDS-PAGE) and transferred onto nitrocellulose membranes (Immobilon-P, Millipore, Zug, Switzerland) at 350 mA for 60 min. Membranes were blocked for 1 h at room temperature with blocking buffer consisting of Tris-buffered saline containing 0.1% Tween-20 (TBS-T) and 5% non-fat dry milk. Membranes were incubated overnight at 4 °C with the following primary antibodies: MFN2 (mouse monoclonal, sc-100560, 1:1000; Santa Cruz Biotechnology, Dallas, TX, USA), DRP1 (mouse monoclonal, sc-3298, 1:1000; Santa Cruz Biotechnology), P62/SQSTM1 (rabbit monoclonal, #5114, 1:1000; Cell Signaling Technology), and LC3B-I/LC3B-II (rabbit monoclonal, #2775, 1:1000; Cell Signaling Technology). GAPDH (mouse monoclonal, sc-32233, 1:1000; Santa Cruz Biotechnology) and alpha-Tubulin (rabbit monoclonal, EP1332Y, 1:1000, ABCAM) were used as a loading control for protein normalization. For autophagy-related markers (p62 and LC3 II/I), membranes were incubated sequentially with the respective antibodies without cropping; therefore, the same α-Tubulin signal was used as a common loading control for both targets.

After incubation with the appropriate horseradish peroxidase-conjugated secondary antibodies, immunoreactive bands were visualized using the iBright 1500 Imaging System (Invitrogen—Thermo Fisher Scientific—Waltham, MA, USA) and quantified by densitometric analysis performed by ImageJ. Data were expressed as fold changes relative to the control group and derived from three independent experiments performed at least in duplicate.

### 2.13. Statistical Analysis

Statistical analyses were performed using GraphPad Prism versin 8.0.2 (GraphPad Software Inc., San Diego, CA, USA). Data were expressed as the mean ± standard deviation (SD) of three independent experiments. Statistical significance was assessed using one-way or two-way analysis of variance (ANOVA), as appropriate, to evaluate the effects of palmitate treatment (500 µM) and onion peel preparations, as specified in the corresponding figure legends. When ANOVA indicated significant differences, post hoc multiple-comparison tests were applied to identify differences between experimental groups. A *p* value < 0.05 was considered statistically significant.

## 3. Results

### 3.1. UHPLC–UV–HRMS/MS Profiling of OPI-T and Comparative Onion Peel Extracts

**Figure 1:**
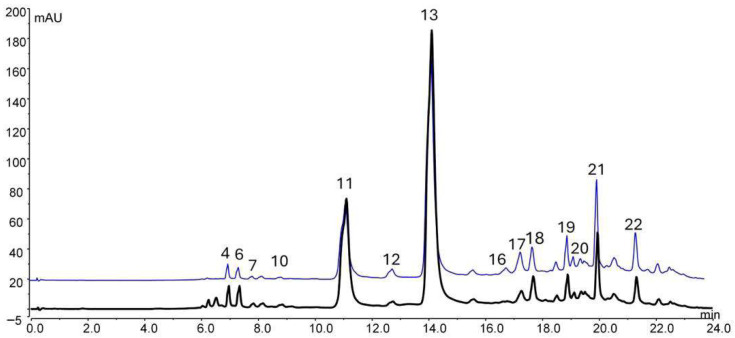
UHPLC-UV profile (365 nm) of Tropea onion skin extract (black) and Montoro onion skin extract (blue).

The UHPLC–UV chromatographic profiles recorded at 365 nm revealed highly comparable phenolic fingerprints for the Tropea (OPI-T) and Montoro onion peel extracts, with differences mainly attributable to relative compound abundance rather than qualitative composition (Figure 1). High-resolution UHPLC–HRMS/MS analysis enabled the tentative identification of 22 phenolic compounds belonging predominantly to the flavonol and anthocyanin classes (Table 1). Quercetin and its mono- and diglycosylated derivatives represented the major constituents detected in both extracts, together with isorhamnetin derivatives, cyanidin glycosides, and higher-order quercetin oligomers. In the Tropea onion peel ingredient (OPI-T), quercetin-based flavonols showed higher signal intensities and a more homogeneous distribution across chromatographic runs, supporting their suitability as marker compounds for ingredient standardization. Anthocyanins, including cyanidin-3-glucoside and malonylated derivatives, were detected primarily in the Tropea extract, consistent with its red cultivar origin. Accurate mass measurements showed mass errors consistently below 5 ppm, and MS/MS fragmentation patterns were fully consistent with reference data previously reported for onion peel phenolics. Overall, the UHPLC–UV–HRMS/MS analysis confirmed that OPI-T maintains a well-defined and reproducible phytochemical profile dominated by quercetin derivatives, supporting its use as a chemically standardized bioactive ingredient for functional and mechanistic studies (Table S1).

### 3.2. OPI-T Does Not Significantly Affect HepG2 Cell Viability at Functionally Relevant Concentrations

The effect of the standardized onion peel bioactive ingredient obtained from *Allium cepa* L. cv. Rossa di Tropea (OPI-T) on HepG2 cell viability was evaluated by MTT assay after 24 and 48 h of treatment at concentrations ranging from 6.25 to 50 µg/mL, using ethanol-treated cells as controls.

**Figure 2:**
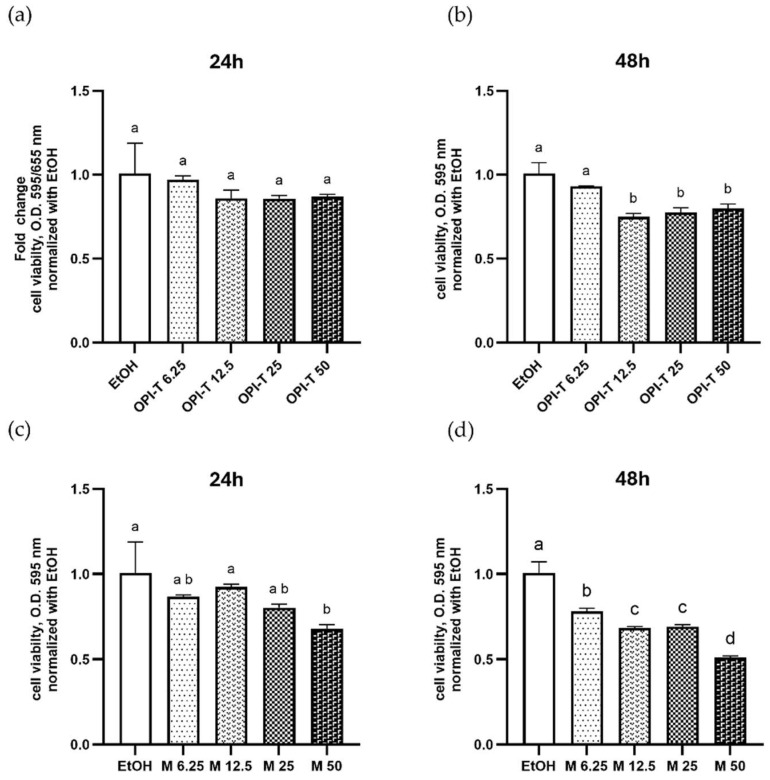
Effect of the standardized onion peel bioactive ingredient (OPI-T) and non-standardized onion peel extract from *Allium cepa* L. cv. Ramata di Montoro (M) on HepG2 cell viability. HepG2 cells were treated with OPI-T and M at concentrations of 6.25, 12.5, 25, and 50 µg/mL for 24 h (**a**,**c**) and 48 h (**b**,**d**). Ethanol-treated cells (EtOH) were used as controls. Cell viability was assessed by MTT assay. Data are expressed as mean ± SD of three independent experiments performed in triplicate. Statistical analysis was performed using one-way ANOVA followed by Tukey’s multiple-comparison test. (**b**) *p* ≤ 0.05 vs. EtOH. Different letters indicate statistically different values (*p* ≤ 0.05).

OPI-T showed a limited and time-dependent effect on cell viability, with no evidence of marked cytotoxicity after 24 h of treatment (Figure 2a,b), whereas after 48 h, an approximately 20% maximum reduction was observed at the doses from 12.5 to 50 µg/mL. These data indicate that OPI-T is well tolerated by HepG2 cells at 24 h-treatment in the dose range used.

For comparison, the non-standardized onion peel extract obtained from *Allium cepa* L. cv. Ramata di Montoro induced a more pronounced reduction in cell viability at comparable concentrations (Figure 2c,d). After 24 h, Montoro extract treatment resulted in a significant decrease in viability at 50 µg/mL (approximately 32%). This effect was further exacerbated after 48 h, with reductions reaching approximately 30% at intermediate doses (12.5 and 25 µg/mL) and nearly 50% at 50 µg/mL.

Taken together, these results demonstrate that OPI-T displays a more favorable tolerability profile than the non-standardized Montoro extract, supporting its selection as a bioactive ingredient suitable for functional studies and further development. Furthermore, the 24 h timing proved to be suitable for subsequent functional studies, due to the absence or low impact of the OPI-T and Montoro extract on cell viability, respectively.

### 3.3. OPI-T Mildly Modulates HepG2 Cell Migration Without Inducing Excessive Antiproliferative Effects

**Figure 3:**
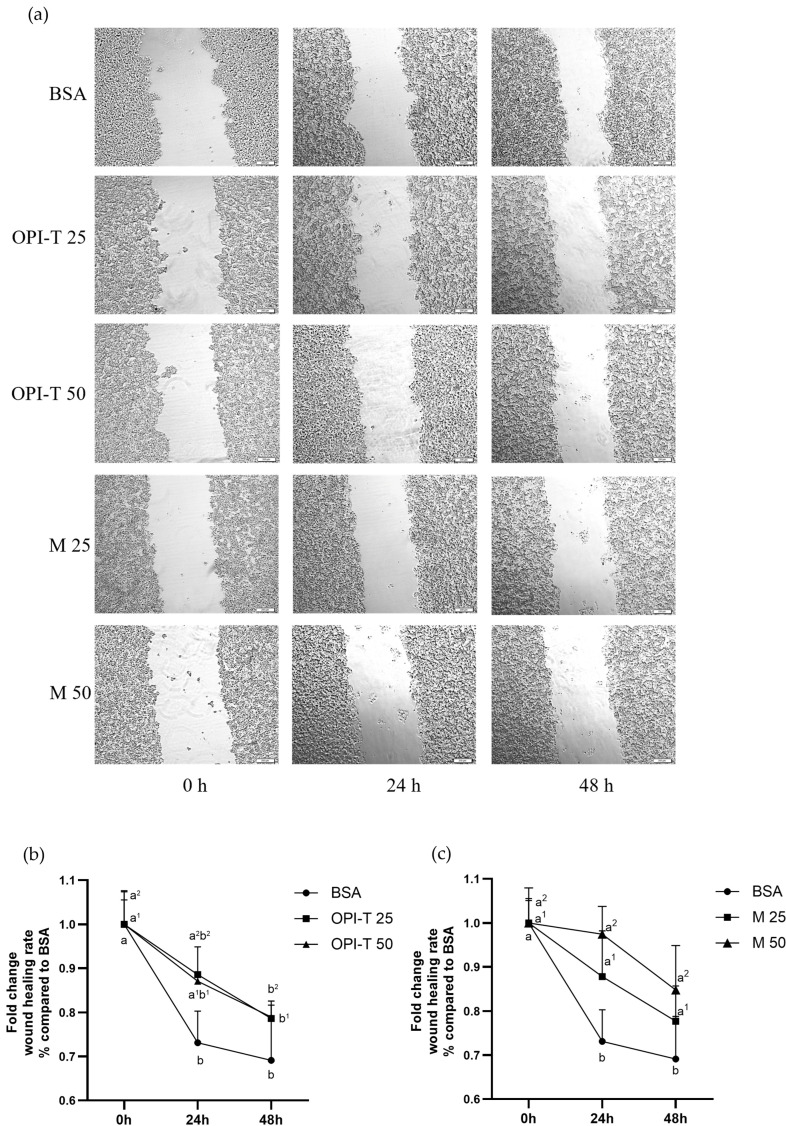
Effect of the standardized onion peel bioactive ingredient (OPI-T) and non-standardized onion peel extract from *Allium cepa* L. cv. Ramata di Montoro (M) on HepG2 cell migration. (**a**) Representative images of wound closure at 0, 24, and 48 h following treatment with BSA (control), OPI-T at 25 and 50 µg/mL, M at 25 and 50 µg/mL. Images were acquired at 20× magnification; scale bar: 200 µm. (**b**) Quantification of wound closure (%) in HepG2 cells treated with OPI-T at 25 and 50 µg/mL after 0, 24 and 48 h. (**c**) Quantification of wound closure (%) in HepG2 cells treated with the Montoro onion peel extract at 25 and 50 µg/mL after 0, 24 and 48 h. Data are expressed as mean ± SD of three independent experiments performed in triplicate. Statistical analysis was performed using two-way ANOVA followed by Tukey’s multiple-comparison test. Two-way ANOVA results: (**b**) significant effect of treatment (*p* = 0.0182), extremely significant effect of time (*p* < 0.0001), no significant interaction (*p* = 0.2618); (**c**) very significant effect of treatment (*p* = 0.0084), extremely significant effect of time (*p* < 0.0001), no significant interaction (*p* = 0.1714). Different letters indicate statistically different values (*p* ≤ 0.05). ^1^ Referred to OPI-T or M at 25 µg/mL and after 0, 24 and 48 h treatment; ^2^ referred to OPI-T or M at 50 µg/mL after 0, 24 and 48 h treatment.

To further characterize the effects of the standardized onion peel bioactive ingredient (OPI-T) on HepG2 cell behavior, a wound healing (scratch) assay was performed to evaluate cell migration following treatment. Control cells exhibited a progressive and significant wound closure over time, with approximately 27% closure after 24 h and about 30% after 48 h, confirming the intrinsic migratory capacity of HepG2 cells under basal conditions (Figure 3a,b).

Treatment with OPI-T resulted in a moderate, time-dependent reduction in cell proliferation with a 20% wound closure after 48 h at the concentrations of 25 and 50 µg/mL (Figure 3a,b). This finding is consistent with the slight decrease in cell viability observed in MTT assays after 48 h. Importantly, wound closure was not completely inhibited at any tested concentration, indicating that OPI-T does not exert a strong antiproliferative or cytotoxic effect on HepG2 cells, but rather induces a controlled modulation of cell migration.

In contrast, the non-standardized onion peel extract obtained from *Allium cepa* L. cv. Ramata di Montoro induced a more pronounced inhibition of wound closure (Figure 3a,c). Treatment with 25 µg/mL and 50 µg/mL of Montoro extract resulted in a minimal wound closure after 24 h and a not significantly 15% closure after 48 h, suggesting a stronger impairment of cell migratory capacity.

In conclusion, these results indicate that OPI-T displays a more balanced biological profile than the non-standardized Montoro extract, characterized by limited modulation of cell migration without excessive antiproliferative effects. This behavior supports the suitability of OPI-T for subsequent functional studies aimed at evaluating its protective activity against palmitate-induced steatosis and oxidative stress.

### 3.4. OPI-T Attenuates Palmitate-Induced Lipid Accumulation in HepG2 Cells

**Figure 4:**
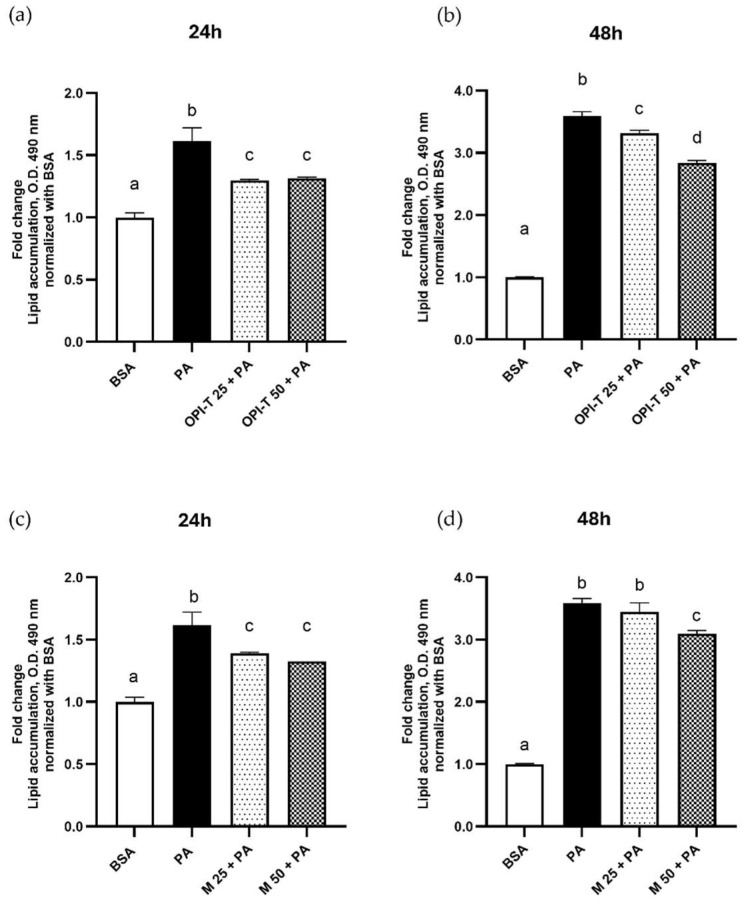
Effect of the standardized onion peel bioactive ingredient (OPI-T) and non-standardized onion peel extract from *Allium cepa* L. cv. Ramata di Montoro (M) on palmitate-induced lipid accumulation in HepG2 cells. Quantitative Oil Red O staining was performed after 24 h (**a**,**c**) and 48 h (**b**,**d**) of treatment with BSA (control), palmitate (PA, 500 µM), or palmitate in combination with OPI-T and M at 25 and 50 µg/mL. Data are expressed as mean ± SD of at least three independent experiments performed in triplicate. Statistical analysis was carried out using one-way ANOVA followed by Tukey’s multiple-comparison test. Different letters indicate statistically different values (*p* ≤ 0.05).

To evaluate the antisteatotic potential of the standardized onion peel bioactive ingredient (OPI-T), intracellular lipid accumulation was assessed by quantitative Oil Red O staining in HepG2 cells exposed to palmitate. Treatment with palmitate (500 µM) induced a marked increase in intracellular lipid content compared to control cells, confirming the establishment of a steatotic phenotype. After 24 h of treatment, palmitate exposure resulted in a significant increase in lipid accumulation (+61% vs. control), as shown in Figure 4a. This effect was significantly attenuated by the simultaneous treatment with OPI-T at both 25 and 50 µg/mL, which reduced lipid accumulation by approximately 18% compared to palmitate alone. After 48 h, palmitate treatment induced a more pronounced steatotic response, with a 3.6-fold increase in lipid content relative to control cells (Figure 4b). Under these conditions, OPI-T significantly counteracted lipid accumulation at the highest concentration tested (50 µg/mL), resulting in an approximate 21% reduction compared to palmitate-treated cells.

**Figure 5:**
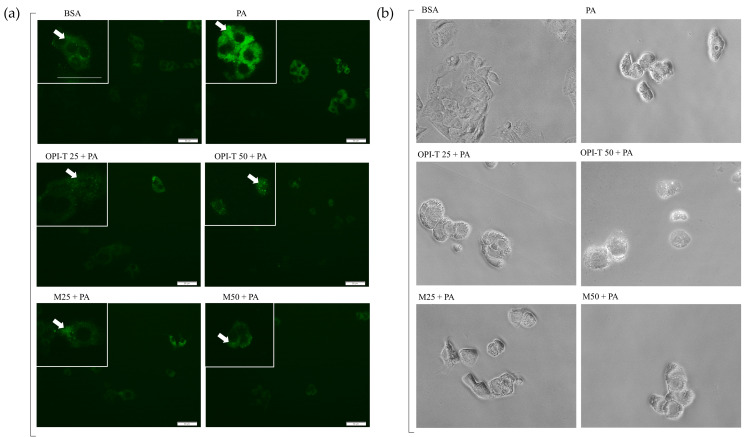
Qualitative assessment of intracellular lipid accumulation and cell morphology in HepG2 cells treated with OPI-T or non-standardized onion peel extract from *Allium cepa* L. cv. Ramata di Montoro (M) under palmitate-induced steatotic conditions. (**a**) Representative BODIPY™ 493/503 fluorescence images showing intracellular lipid droplets after 24 h of treatment with BSA (control), palmitate (PA, 500 µM), or palmitate in combination with OPI-T or M at 25 and 50 µg/mL. White arrows indicate intracellular lipid droplets. (**b**) Representative phase-contrast images illustrating cell morphology under the same experimental conditions. Images were acquired at 20× magnification; scale bar: 50 µm.

For comparison, the non-standardized onion peel extract obtained from *Allium cepa* L. cv. Ramata di Montoro also partially attenuated palmitate-induced lipid accumulation, although with a less consistent profile (Figure 4c,d). After 24 h, both tested concentrations (25 and 50 µg/mL) induced a modest reduction (≈15%) in lipid content compared to palmitate alone (Figure 4c). After 48 h, a significant decrease (≈14%) was observed only at the highest concentration (50 µg/mL) (Figure 4d). Qualitative analysis of intracellular lipid droplets by BODIPY fluorescence staining further supported the quantitative Oil Red O results (Figure 5a). Palmitate treatment markedly increased fluorescence intensity and was associated with evident morphological alterations, including cell rounding and cytoplasmic lipid droplet accumulation (Figure 5b). Co-treatment with OPI-T and Montoro extract resulted in a clear reduction in intracellular lipid fluorescence at both tested concentrations, while palmitate-induced morphological alterations were not recovered.

Taken together these results demonstrate that OPI-T effectively attenuates palmitate-induced lipid accumulation in HepG2 cells, with a more robust and coherent antisteatotic profile than the non-standardized Montoro extract. This finding supports the role of standardization in enhancing the functional efficacy of onion peel-derived bioactive ingredients.

### 3.5. OPI-T Markedly Reduces Palmitate-Induced Oxidative Stress in HepG2 Cells

**Figure 6:**
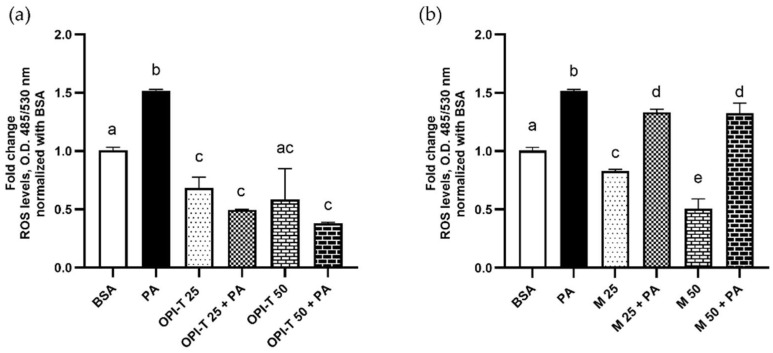
Effect of the standardized onion peel bioactive ingredient (OPI-T) and non-standardized onion peel extract from *Allium cepa* L. cv. Ramata di Montoro (M) on intracellular ROS production under palmitate-induced oxidative stress in HepG2 cells after 24 h. (**a**) ROS levels were measured by DCF assay after 24 h of treatment with BSA (control), palmitate (PA, 500 µM), OPI-T alone (25 or 50 µg/mL), or OPI-T in combination with PA. Data are expressed as mean ± SD of three independent experiments performed in triplicate. Statistical analysis was carried out using two-way ANOVA followed by Tukey’s multiple-comparison test. Two-way ANOVA results: extremely significant effect of OPI-T dose (*p* < 0.0001), n.s. effect of palmitate (*p* = 0.4701), and highly significant interaction (*p* = 0.0002). Different letters indicate statistically different values. (**b**) ROS levels were measured by DCF assay after 24 h of treatment with BSA (control), palmitate (PA, 500 µM), M alone (25 or 50 µg/mL), or M in combination with palmitate. Data are expressed as mean ± SD of three independent experiments performed in triplicate. Statistical analysis was carried out using two-way ANOVA followed by Tukey’s multiple-comparison test. Two-way ANOVA results: extremely significant effect of OPI-T dose (*p* < 0.0001), extremely significant effect of palmitate (*p* < 0.0001) and highly significant interaction (*p* = 0.0002). Different letters indicate statistically different values (*p* ≤ 0.05).

To investigate the effects of the standardized onion peel bioactive ingredient (OPI-T) on oxidative stress, intracellular reactive oxygen species (ROS) levels were measured after 24 h of treatment using the DCF assay. Exposure of HepG2 cells to palmitate (500 µM) resulted in a significant increase in ROS production (+52% vs. control), confirming the establishment of an oxidative stress condition associated with lipid overload (Figure 6a,b).

Treatment with OPI-T alone significantly reduced basal ROS levels in a dose-dependent manner. In particular, ROS production was reduced by approximately 30% and 41% at concentrations of 25 and 50 µg/mL, respectively, compared to that of control cells (Figure 6a). Notably, the co-treatment with OPI-T and palmitate resulted in a marked attenuation of palmitate-induced oxidative stress, with reductions of approximately 67% and 75% at 25 and 50 µg/mL, respectively, compared to palmitate alone (Figure 6a). Two-way ANOVA revealed an extremely significant effect of OPI-T dose (*p* < 0.0001), a non-significant main effect of palmitate (*p* = 0.4701), and a highly significant interaction between the two factors (*p* = 0.0002), indicating that the antioxidant effect of OPI-T is strongly dependent on its concentration and is particularly evident under lipotoxic conditions, showing an antagonistic interaction with palmitate.

For comparison, the non-standardized onion peel extract obtained from *Allium cepa* L. cv. Ramata di Montoro also reduced intracellular ROS levels when administered alone, with decreases of approximately 17% and 49% at 25 and 50 µg/mL, respectively (Figure 6b). However, when combined with palmitate, the Montoro extract produced only a modest attenuation of oxidative stress, with reductions of approximately 12% at both tested concentrations compared to palmitate alone (Figure 6b). In this case, two-way ANOVA showed an extremely significant effect of both treatment (*p* < 0.0001) and palmitate (*p* < 0.0001), together with a highly significant interaction (*p* = 0.0002), with antagonistic effect between palmitate and extract. The results suggest a less selective and less effective antioxidant response under lipotoxic conditions compared to OPI-T extracts.

These results demonstrate that OPI-T exerts a robust and dose-dependent antioxidant effect in HepG2 cells, particularly under palmitate-induced oxidative stress. The markedly greater efficacy of OPI-T compared to the non-standardized Montoro extract further supports the relevance of chemical standardization in enhancing the functional performance of onion peel-derived bioactive ingredients.

### 3.6. OPI-T Modulates Mitochondrial Dynamics by Restoring the Fusion–Fission Balance Under Palmitate-Induced Stress

Given the central role of mitochondrial dynamics in cellular adaptation to metabolic stress, the effects of the standardized onion peel bioactive ingredient (OPI-T) on mitochondrial fusion and fission processes were investigated by evaluating the protein levels of mitofusin-2 (MFN2) and dynamin-related protein-1 (DRP1) after 24 h of treatment at the highest concentration tested (50 µg/mL).

**Figure 7:**
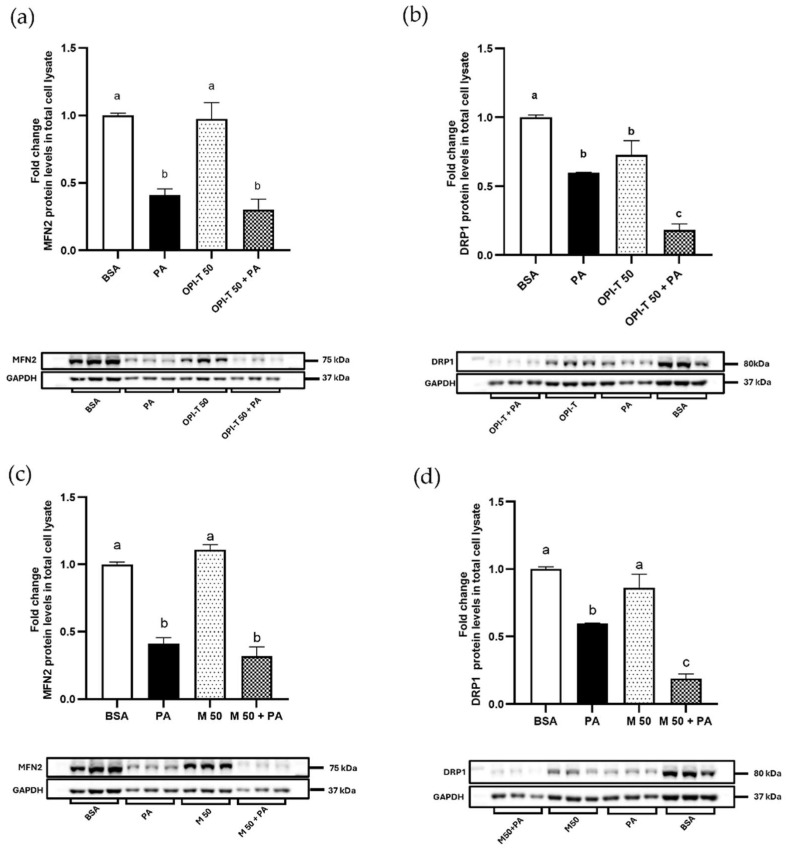
Effect of the standardized onion peel bioactive ingredient (OPI-T) and non-standardized onion peel extract from *Allium cepa* L. cv. Ramata di Montoro (M) on mitochondrial fusion and fission markers under palmitate-induced stress in HepG2 cells. Representative Western blot and densitometric analysis of MFN2 and DRP1 protein levels normalized to GAPDH in HepG2 cells treated for 24 h with (**a**,**b**) BSA (control), palmitate (PA, 500 µM), OPI-T alone (50 µg/mL), or OPI-T in combination with palmitate (**c**,**d**) BSA (control), palmitate (PA, 500 µM), M alone (50 µg/mL), or M in combination with palmitate. Data were expressed as means ± SD of three independent experiments performed at least in duplicate. Statistical analysis was carried out using two-way ANOVA followed by Tukey’s multiple-comparison test. Two-way ANOVA results: (**a**) n.s. significant effect of OPI-T (*p* = 0.1701), extremely significant effect of palmitate (*p* < 0.0001), n.s. significant interaction (*p* = 0.3856); (**b**) extremely significant effect of OPI-T (*p* < 0.0001), extremely significant effect of palmitate (*p* < 0.0001), n.s. significant interaction (*p* = 0.06); (**c**) n.s. significant effect of non-standardized onion peel extract from *Allium cepa* L. cv. Ramata di Montoro (M) (*p* = 0.7210), extremely significant effect of palmitate (*p* < 0.0001), very significant interaction between the two factors (*p* = 0.0056); (**d**) extremely significant effect of non-standardized onion peel extract from *Allium cepa* L. cv. Ramata di Montoro (M) (*p* < 0.0001), extremely significant effect of palmitate (*p* < 0.0001), very significant interaction (*p* = 0.0025). Different letters indicate statistically different values (*p* ≤ 0.05).

Treatment with palmitate alone induced a marked alteration of mitochondrial dynamics, characterized by a significant reduction in MFN2 protein content (−59%) (Figure 7a,c) and a concomitant decrease in DRP1 levels (−40%) (Figure 7b,d) compared to control cells. As a consequence, the MFN2/DRP1 ratio was significantly reduced (0.68 vs. 1.00 in control cells; −32%), indicating a shift toward a mitochondrial fission-prone state.

OPI-T administered alone did not significantly affect MFN2 levels compared to control cells, while it induced a significant reduction in DRP1 content (−28%) (Figure 7a,b). This resulted in a significant increase in the MFN2/DRP1 ratio (1.34; +34% vs. control), suggesting a shift toward mitochondrial fusion under basal conditions.

Notably, co-treatment with OPI-T and palmitate profoundly modified the mitochondrial response to lipid overload. In these conditions, DRP1 protein levels were strongly reduced (−70% vs. palmitate alone) (Figure 7b), while MFN2 levels were only moderately decreased (−26% vs. palmitate alone) (Figure 7a). As a result, the MFN2/DRP1 ratio markedly increased (1.66), exceeding both control and palmitate-treated cells and indicating a robust restoration of a fusion-favored mitochondrial phenotype.

For comparison, the non-standardized onion peel extract obtained from *Allium cepa* L. cv. Ramata di Montoro showed a similar modulation of mitochondrial dynamics. Treatment with the Montoro extract alone induced minor changes in MFN2 content (+11%) and a moderate decrease in DRP1 levels (−14%), resulting in an increase in the MFN2/DRP1 ratio (1.29; +29% vs. control) (Figure 7c,d). Under palmitate co-treatment, both MFN2 and DRP1 protein levels were reduced (−22% and −68% vs. palmitate alone, respectively), leading to an increase in the MFN2/DRP1 ratio (1.68) compared to palmitate alone.

### 3.7. OPI-T Modulates Autophagy-Related Markers in Palmitate-Treated HepG2 Cells

To further investigate the cellular adaptive response to palmitate-induced stress, the involvement of autophagy was evaluated by analyzing the protein levels of p62 and the LC3-II/LC3-I ratio, two widely used markers associated with autophagic processes.

**Figure 8:**
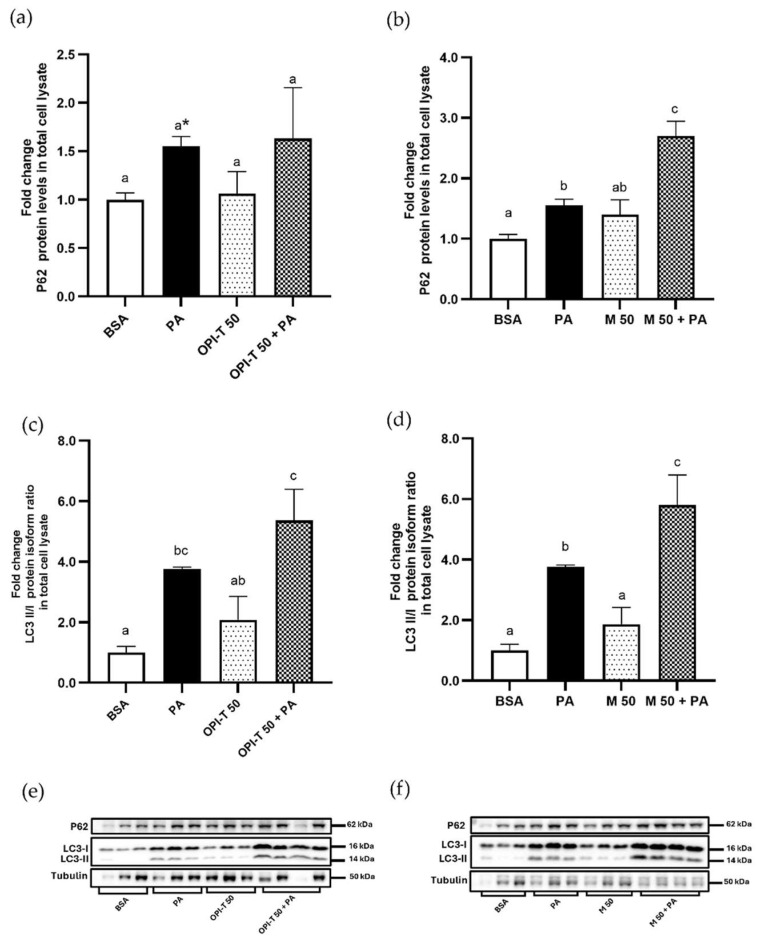
Effect of the standardized onion peel bioactive ingredient (OPI-T) and non-standardized onion peel extract from *Allium cepa* L. cv. Ramata di Montoro (M) on mitochondrial fusion and fission markers under palmitate-induced stress in HepG2 cells. Representative Western blot and densitometric analysis of P62 and LC3 II/I ratio protein levels normalized to Tubulin in HepG2 cells treated for 24 h with (**a**,**c**,**e**) BSA (control), palmitate (PA, 500 µM), OPI-T alone (50 µg/mL), or OPI-T in combination with palmitate and (**b**,**d**,**f**) BSA (control), palmitate (PA, 500 µM), M alone (50 µg/mL), or M in combination with palmitate. Data are expressed as means ± SD derived from three independent experiments performed at least in duplicate. Statistical analysis was carried out using two-way ANOVA followed by Tukey’s multiple-comparison test. Two-way ANOVA results: (**a**) non-significant effect of OPI-T (*p* = 0.6857), significant effect of palmitate (*p* = 0.0103), n.s. interaction (*p* = 0.9602). Student’s *t*-test: * *p* = 0.0014 PA vs. BSA; (**b**) extremely significant effect of M (*p* < 0.0001), extremely significant effect of palmitate (*p* < 0.0001), very significant interaction between the two factors (*p* = 0.0077); (**c**) very significant effect of OPI-T (*p* = 0.0075), extremely significant effect of palmitate (*p* < 0.0001), n.s. significant interaction (*p* = 0.5170); (**d**) very significant effect of M (*p* = 0.0024), extremely significant effect of palmitate (*p* < 0.0001), n.s. interaction (*p* = 0.1185). Different letters indicate statistically different values (*p* ≤ 0.05).

Palmitate treatment alone induced a significant increase in p62 protein levels (+55%) compared to control cells (Figure 8a,b), together with a marked elevation of the LC3-II/LC3-I ratio (approximately 3.8-fold), indicating a modulation of autophagy-related pathways under lipotoxic conditions (Figure 8c,d).

Treatment with the standardized Tropea red onion peel ingredient (OPI-T) alone or combined with palmitate did not significantly affect p62 levels compared to control or palmitate treated cells, respectively (Figure 8a). Notably, OPI-T increased the LC3-II/LC3-I ratio, with a two-fold elevation in extract-treated cells and a further enhancement upon co-treatment with palmitate (5.4-fold vs. control and +40% vs. palmitate alone), suggesting a potentiation of autophagy-associated signaling under lipotoxic stress (Figure 8c).

In contrast, the non-standardized Montoro coppery onion peel extract exhibited a stronger modulation of autophagy markers in palmitate induced stress condition. The extract alone increased p62 protein levels (+40% vs. control), while co-treatment with palmitate resulted in a pronounced accumulation of p62 (2.7-fold vs. control and +74% vs. palmitate alone). The two-way ANOVA statistical analysis highlighted a very significant interaction between the two factors, with a synergistic effect in increasing P62 content (Figure 8b). Similarly, the LC3-II/LC3-I ratio was increased by the Montoro extract alone (+87% vs. control) and was further enhanced when combined with palmitate (approximately 6-fold vs. control and +54% vs. palmitate alone) (Figure 8d).

These findings indicate that OPI-T induces a controlled modulation of autophagy-related markers, particularly under palmitate-induced stress, whereas the non-standardized Montoro extract elicits a more pronounced and less regulated activation of autophagy-associated responses.

## 4. Discussion

Fatty liver disease, due to its high prevalence and possible progression to more severe forms of liver disease, represents a significant challenge to public health and therefore the need for ways to prevent or treat such diseases is urgently needed [1,2]. The present study investigated the protective effect of Tropea and Montoro onion peel extracts on lipid accumulation, oxidative stress, mitochondrial dynamics and autophagy in an in vitro cellular model of fatty liver disease. In vitro hepatocyte models represent a fundamental tool for studying the cellular mechanisms underlying the etiopathogenesis of hepatic steatosis and MASLD. Ethical issues and limited availability of human liver samples complicate the use of primary human cell cultures in studies [16,34,43]. Consequently, the HepG2 cell line, derived from hepatoblastoma, represents a practical and reliable alternative for these types of studies [29,30,31,32,33,34,35,36]. Despite their tumor origin, they retain many structural and functional features of normal human hepatocytes, including lipid handling and glucose metabolism, which makes them especially suitable for studying both cancerous and non-cancerous liver disorders [11,12,13,14,15,16,17,18]. Their immortalized nature, stable phenotype, and high reproducibility further strengthen their value as a reliable in vitro system. Therefore, in the present study, HepG2 cells were used to generate an in vitro model of fatty acid-induced steatosis to investigate the effects of onion peel extracts. In particular, treatment with palmitic acid (PA)—the most abundant saturated fatty acid in both healthy and MASLD conditions—reliably induces lipid accumulation and metabolic dysfunction in these cells, allowing the establishment of robust and reproducible MASLD models [10,11,12,13,14,15,16,17,18,30,34]. Using this approach, the present study demonstrated that both Tropea and Montoro onion peel extracts are able to attenuate palmitate-induced steatosis in HepG2 cells, in association with a reduction in oxidative stress and a coordinated modulation of mitochondrial dynamics and autophagy-related pathways. The dosage and timing of treatment with onion peel extracts were chosen to ensure low or no cytotoxic effects, antioxidant effects, as well as greater than 10% attenuation of palmitate-induced lipid accumulation for both extracts.

The dose-dependent effects of Tropea and Montoro onion peel extracts on cell viability and oxidative stress were initially evaluated by MTT and DCF assays, respectively. In a previous study, the antioxidant properties of the same extracts were assessed using chemical assays such as ABTS and oxygen radical absorbance capacity (ORAC) [8]. While these assays are valuable for characterizing redox activity in vitro, they do not fully reflect the complexity of antioxidant behavior in a cellular context. Indeed, within biological systems, antioxidants act not merely by scavenging free radicals, but by preserving redox homeostasis and protecting cellular structures from oxidative damage. For this reason, the present study focused on evaluating the antioxidant effects of onion peel extracts directly in a hepatic cellular model.

Both extracts significantly reduced intracellular ROS levels in HepG2 cells at the doses of 25 and 50 µg/mL after 24 h of treatment, confirming and extending the antioxidant activity previously observed in chemical assays. In parallel, at the same dose range and timing indicated, OPI-T extracts did not induce significant cytotoxic effects, whereas Montoro extract induced a 32% decrease in cell viability at the dose of 50 µg/mL after 24 h of treatment. This observation differs from the findings of Celano et al. [8], where no cytotoxic effects were observed in human fibroblasts and, in some cases, cell proliferation was enhanced. This apparent discrepancy can be explained by considering both the differences in the dose range and timing used and the intrinsic differences between the two cell models. HepG2 cells are hepatocellular carcinoma cells, whereas fibroblasts represent non-transformed cells. Therefore, the reduction in viability observed in HepG2 cells likely reflects an antiproliferative rather than a cytotoxic effect.

This interpretation is supported by previous studies reporting growth inhibition of cancer cells following treatment with onion peel extracts, including HT-29 colorectal cancer cells [44]. To further explore this possibility, cell migration was evaluated using a wound healing assay. The reduced wound closure observed in extract-treated cells indicates a slowdown of cell migration and proliferation, supporting the hypothesis that the decrease in cell viability reflects an antiproliferative effect. Importantly, this effect was moderate and did not result in complete inhibition of cell migration, particularly for the standardized Tropea extract (OPI-T).

Beyond the modulation of cell proliferation, cells treated with onion peel extracts exhibited a reduction in ROS production and shift in mitochondrial dynamics toward fusion, which is generally associated with improved mitochondrial function, thus suggesting a role of mitochondrial function in the maintenance of cellular homeostasis [20,23,27,38,41].

To evaluate the antisteatotic potential of onion peel extracts, HepG2 cells were rendered steatotic using palmitate (500 µM). This treatment induced a time-dependent accumulation of intracellular lipids, as demonstrated by both quantitative Oil Red O analysis and qualitative BODIPY fluorescence imaging. Lipid accumulation was accompanied by increased ROS production and a shift toward mitochondrial fission, as indicated by a reduced MFN2/DRP1 ratio. These findings are consistent with previous studies showing that palmitate induces mitochondrial fragmentation and oxidative stress in HepG2 cells, with mitochondrial alterations preceding ROS generation [44,45,46]. Moreover, excessive mitochondrial fission has been associated with the development of MAFLD, as well as with increased oxidative stress [21,22,23,24].

Palmitate treatment also impaired autophagy-related processes, as indicated by the concomitant increase in LC3-II/LC3-I ratio and p62 accumulation. This pattern is consistent with an inhibition of autophagic flux, as previously reported by Kim et al., 2025 [47]. Autophagy modulation in response to palmitate appears to be time-dependent: initial activation may serve as a compensatory mechanism to limit lipid overload [47,48,49], whereas prolonged exposure, as in the present study (24 h), leads to autophagy dysfunction.

The main objective of this study was to assess whether onion peel extracts could counteract palmitate-induced steatosis. Both quantitative and qualitative analyses demonstrated that simultaneous treatment with palmitate and either extract significantly reduced intracellular lipid accumulation. This antisteatotic effect was slightly more pronounced for the Tropea onion peel extract compared to the Montoro extract. Notably, the antioxidant effect observed under co-treatment conditions was also greater for the Tropea extract, in line with its higher antioxidant capacity reported previously using chemical assays [8]. These differences are likely attributable to variations in the chemical composition of the two extracts. The biological activity of plant-derived extracts is strongly influenced by their chemical standardization, which is increasingly recognized as a prerequisite for reproducibility and translational relevance in nutraceutical research. Several studies have demonstrated that standardized polyphenol-rich extracts, characterized by defined marker compounds and controlled batch-to-batch variability, exert more consistent metabolic and hepatoprotective effects than non-standardized preparations [49,50,51]. Standardized extracts of green tea, milk thistle, and citrus polyphenols have shown improved efficacy in modulating oxidative stress, lipid accumulation, and mitochondrial function in hepatic models when marker compounds and quality specifications are defined [52,53,54,55,56,57,58]. In this context, the more consistent biological profile observed for the standardized Tropea onion peel ingredient (OPI-T), compared to the non-standardized Montoro extract, aligns with current evidence supporting standardization as a key determinant of functional reliability.

Our findings support the protective role of polyphenol-rich extracts against lipotoxic stress and are consistent with recent evidence indicating that dietary polyphenols modulate both lipid metabolism and redox homeostasis in hepatic models. In particular, phenolic compounds have been shown to counteract oxidative stress and improve metabolic resilience through the regulation of antioxidant defenses and mitochondrial function [59,60,61,62].

In terms of mitochondrial dynamics, both extracts attenuated the palmitate-induced shift toward mitochondrial fission, as evidenced by an increased MFN2/DRP1 ratio compared to palmitate-treated cells. Since mitochondrial fusion is associated with improved mitochondrial function and bioenergetic efficiency, whereas excessive fission is linked to mitochondrial damage and mitophagy, the ability of onion peel extracts to restore the fusion–fission balance may contribute to the observed reduction in oxidative stress and lipid accumulation. Consistent with our findings, recent studies have demonstrated that natural phenolic compounds, particularly ferulic acid (major phenolic compound found in whole grains), can restore mitochondrial dynamics in palmitate-exposed hepatocyte models, contributing to improved cellular homeostasis and metabolic function [63].

Regarding autophagy, distinct patterns emerged between the two extracts. In cells co-treated with palmitate and the Tropea extract, p62 levels were not further increased compared to palmitate alone, while the LC3-II/LC3-I ratio was enhanced. This suggests that autophagy-related processes were activated without excessive accumulation of autophagosomes. In contrast, co-treatment with the Montoro extract resulted in increased levels of both p62 and LC3-II/LC3-I ratio, indicating a stronger activation of early autophagy-related events. This response may reflect an attempt by the cell to counteract lipid overload by enhancing autophagosome formation, particularly during the initial stages of autophagy in which p62 plays a key role [64]. However, it is important to emphasize that LC3 and p62 measurements alone are not sufficient to conclusively assess autophagic flux. Indeed, the accumulation of LC3-II may reflect either enhanced autophagosome formation or impaired degradation, while p62 levels can increase due to reduced autophagic clearance or transcriptional upregulation. Therefore, our data should be interpreted as indicative of autophagy modulation rather than definitive activation of autophagic flux, in line with current recommendations in the field [64].

Overall, the modulation of autophagy-related markers observed under co-treatment conditions is consistent with the reduction in lipid accumulation and oxidative stress, suggesting that autophagy may contribute to the cellular adaptive response aimed at removing lipid droplets and damaged organelles. Similar mechanisms have been described for bioactive compounds such as apigenin and epigallocatechin gallate, which promote lipid droplet degradation through autophagy modulation [65,66,67]. Moreover, recent studies have also shown that quercetin, of which onion peel is a rich source, can modulate autophagy and reduce hepatic steatosis, partly through the regulation of LC3 and p62 and the improvement of metabolic parameters in liver disease models [68]. Moreover, quercetin has been reported to attenuate inflammatory responses and regulate autophagy-related pathways in hepatic systems [69]. Beyond quercetin [70] other polyphenols such as resveratrol [6,47,67] and ferulic acid [63] have been shown to exert similar protective effects in metabolic and hepatic contexts, including the reduction in lipid accumulation, attenuation of oxidative stress, and modulation of mitochondrial function/dynamics and autophagy-related pathways, reinforcing the concept of a shared mechanistic framework among different classes of polyphenols [63].

Nevertheless, further experiments specifically addressing autophagic flux are required to clarify the precise role of autophagy in the antisteatotic effects of onion peel extracts; the lack of direct assessment of autophagic flux (e.g., using lysosomal inhibitors) limits the interpretation of autophagy-related findings.

In conclusion, Tropea and Montoro onion peel extracts exert protective effects against palmitate-induced steatosis in HepG2 cells by reducing oxidative stress, improving mitochondrial dynamics, and modulating autophagy-related pathways. Further analyses are needed to confirm the superior bioactivity of OPI-T compared to Montoro extract. However, these findings support the potential use of onion peel-derived bioactive ingredients as functional agents for the prevention or management of hepatic steatosis and MAFLD-associated metabolic disorders. Further studies are warranted to elucidate the molecular mechanisms underlying these effects involved and to validate these effects in more complex experimental systems, as well as to identify the specific compounds responsible for the observed biological activities.

## 5. Conclusions

This study demonstrates that onion peel-derived bioactive extracts, particularly the standardized Tropea onion peel ingredient (OPI-T), effectively counteract palmitate-induced steatosis in HepG2 cells by reducing intracellular lipid accumulation and oxidative stress while promoting a coordinated modulation of mitochondrial dynamics and autophagy-related pathways. The use of a chemically standardized extract allowed a more controlled and reproducible biological response compared with a non-standardized reference extract, highlighting the relevance of ingredient standardization for functional and nutraceutical applications. Mechanistically, the protective effects observed appear to be associated with attenuation of mitochondrial fragmentation, partial restoration of redox homeostasis, and adaptive activation of autophagy, suggesting a multifactorial cellular response to lipid overload.

Some limitations of the present study should be acknowledged. The experiments were conducted in a single in vitro hepatocellular carcinoma model, which, although widely used for metabolic and steatosis studies, does not fully recapitulate the complexity of liver physiology in vivo. Palmitate-induced lipotoxicity in HepG2 cells is widely used to model hepatic steatosis, as it promotes lipid accumulation, oxidative stress, and apoptosis [38,71]. However, this model may overestimate lipotoxic effects due to the tumor-derived nature of HepG2 cells. More physiologically relevant systems, including primary hepatocytes and in vivo models, better reflect metabolic complexity but introduce variability and systemic influences [16]. Therefore, although HepG2 cell-based models are valuable for understanding the mechanisms and conducting preliminary assessments, further studies using primary hepatocytes or animal models of diet-induced MASLD or clinical studies are needed to confirm these findings in vivo. In addition, autophagy was assessed through static markers, and future studies addressing autophagic flux dynamics and downstream lipid catabolism pathways will be necessary to further clarify the mechanisms involved. Despite these limitations, the results provide strong proof-of-concept evidence supporting the potential of standardized onion peel-derived ingredients as functional agents for the prevention or mitigation of hepatic steatosis and MASLD-related metabolic alterations in in vitro cellular models. However, the translational relevance is limited and needs to be confirmed by studies in in vivo models and/or clinical trials. Therefore, further in vivo investigations and formulation-oriented studies will be essential to translate these findings toward practical nutraceutical or functional food applications.

## Figures and Tables

**Table 1:** UHPLC-HRMS/MS data of detected compounds in Montoro (M) and Tropea (T) onion dried skin extracts.

				[M − H]^−^ (*m*/*z*)			[M + H]^+^ (*m*/*z*)			
N°	Compound	Molecular Formula	RT_(UV)_ (min)	Measured (*m*/*z*)	Error (ppm)	Product Ion MS/MS	Measured (*m*/*z*)	Error (ppm)	Product Ion MS/MS	Onion
**1**	Protocatechuic acid	C_7_H_6_O_4_	1.0	153.1234	0.9	/	/	/	/	M, T
**2**	2-(3,4-Dihydroxybenzoyl)-2,4,6-trihydroxy-3(2H)-benzofuranone	C_15_H_10_O_8_	6.0	317.0302	1.2	299; 191; 207; 273	/	/	/	M, T
**3**	Cyanidin 3-glucoside	C_21_H_21_O_11_	6.5	/	/	/	449.1076	−0.7	287	T
**4**	Quercetin dihexoside	C_27_H_30_O_17_	7.0	625.1402	0.7	463; 301	627.1524	0.8	465; 303	M, T
**5**	Cyanidin 3-laminaribioside	C_27_H_31_O_16_	6.7	/	/	/	611.1611	0.8	287	T
**6**	Quercetin 3,4′-diglucoside	C_27_H_30_O_17_	7.4	625.1411	0.4	463; 301	627.1533	3.2	465; 303	M, T
**7**	Isorhamnetin dihexoside	C_28_H_32_O_17_	7.9	639.1570	0.03	477; 315	641.1688	3.6	317; 479	M, T
**8**	Cyanidin 3-malonilglucoside	C_24_H_23_O_14_	8.1	/	/	/	535.1085	0.6	287	T
**9**	Cyanidin 3-malonillaminaribioside	C_30_H_33_O_19_	8.6	/	/	/	697.1605	0.7	287	T
**10**	Quercetin-3-glucoside	C_21_H_20_O_12_	8.9	463.0873	0.6	301	465.1004	3.4	303	M, T
**11**	Quercetin-4′-glucoside	C_21_H_20_O_12_	11.1	463.0872	0.6	301	465.1005	3.9	303	M, T
**12**	Isorhamnetin-O-hexoside	C_22_H_22_O_12_	12.7	477.1030	0.6	315	479.1160	4.3	317	M, T
**13**	Quercetin	C_15_H_10_O_7_	14.1	301.0351	0.9	179; 151	303.0486	3.6	285; 257; 229;	M, T
**14**	Protocatecoyl quercetin	C_22_H_14_O_11_	14.8	453.0452	0.3	299	455.5880	−4.3	437; 301;	M, T
**15**	Protocatecoyl quercetin	C_22_H_14_O_11_	14.9	453.0452	0.6	299	455.0589	−4.6	437; 301;	M, T
**16**	Kaempferol	C_15_H_10_O_6_	16.9	285.0399	2.1	/	287.0535	−4.9	/	M, T
**17**	Isorhamnetin	C_16_H_12_O_7_	17.2	315.0501	0.8	300; 257	317.0640	−3.4	302, 285; 257	M, T
**18**	Quercetin dimer 4′-glucoside	C_36_H_28_O_19_	17.7	763.1142	0.4	611; 449;	765.1259	−4.4	603; 451	M, T
**19**	Quercetin dimer 4′-glucoside	C_36_H_28_O_19_	18.8	763.1140	0.5	611; 600; 299	765.1255	−4.2	603; 585	M, T
**20**	Quercetin dimer hexoside	C_36_H_28_O_19_	19.1	763.1140	0.5	611; 600; 299	765.1265	−4.3	603; 585	M, T
**21**	Quercetin dimer	C_30_ H_18_ O_14_	20.0	601.0615	0.9	449; 299	603.0733	−5.1	585; 313; 303	M, T
**22**	Quercetin trimer	C_45_ H_26_ O_21_	21.3	901.0880	−0.4	299; 449; 599; 601	903.0233	−4.9	885; 751; 585; 613	M, T

## Data Availability

The original contributions presented in the study are included in the article/Supplementary Materials, further inquiries can be directed to the corresponding authors.

## Supplementary Materials

The following supporting information can be downloaded at https://www.mdpi.com/article/10.3390/antiox15040513/s1. Table S1: Flavonoid and anthocyanin content levels in OPI-T; Figure S1: Full unedited western blotting gels for Figure 7a–d; Figure S2: Full unedited western blotting gels for and Figure 8e,f.
