# Supplementary material for: A Standardized Onion Peel-Derived Bioactive Ingredient Attenuates Palmitate-Induced Steatosis and Oxidative Stress by Modulating Mitochondrial Dynamics and Autophagy in HepG2 Cells
Source: Antioxidants (Basel). 2026 Apr 21;15(4):513. doi: 10.3390/antiox15040513 (PMC13113073; doi:10.3390/antiox15040513)
Supplement: Supplementary file 1 [file antioxidants-15-00513-s001.zip › antioxidants-4202940-supplementary materials.pdf]

Supplementary Materials

A standardized onion peel–derived bioactive ingredient attenuates palmitate-induced steatosis and oxidative stress by modulating mitochondrial dynamics and autophagy in HepG2 cells

Ilaria Di Gregorio[1,2], Vincenzo Migliaccio[1], Maria D'Elia, [2,3,4], Rita Celano[2,3], Valentina Santoro[2,3], Anna Lisa Piccinelli[2,3], Maria Teresa Russo[5], Luca Rastrelli[2,3]*, Lillà Lionetti[1,3]*

[1] Department of Chemistry and Biology "A. Zambelli", University of Salerno, Via Giovanni Paolo II, 132, 84084, Salerno, Italy

[2] Department of Pharmacy, University of Salerno, Via Giovanni Paolo II, 132, 84084, Salerno, Italy

[3] National Biodiversity Future Center (NBFC), 90133 Palermo, Italy;

[4] Dipartimento di Scienze della Terra e del Mare, University of Palermo. Palermo, Italy

[5] Department of Agriculture Science, Food Chemistry, Safety and Sensoromic Laboratory (FoCuSS Lab), University of Reggio Calabria, Via Salita Melissari, 89124 Reggio Calabria, Italy;

* Correspondence: rastrelli@unisa.it (L.R.); llionetti@unisa.it (L.L.)

Table S1 Flavonoid and anthocyanin content levels in OPI-T

OPI-T	Exhaustive Extract (mg/g)
Cyanidin 3-glucoside	21.9 ± 1.7
Cyanidin 3-laminaribioside[a]	1.7 ± 0.4
Quercetin 3,4'-diglucoside[b]	1.9 ± 0.7
Quercetin-4'-glucoside[b]	26.9 ± 2.1
Quercetin	68.6 ± 3.1
Quercetin dimer[b]	7.5 ± 1.5
Quercetin dimer 4'-glucoside[b]	3.2 ± 0.6
Quercetin dimer 4'-glucoside[b]	3.1 ± 0.7
Quercetin trimer[b]	3.5 ± 0.9

[a] expressed as cyanidin 3 glucoside equivalents; [b] expressed as quercetin equivalents

Quantitative analyses of onion skin extracts were performed using a Dionex Ultimate 3000 UHPLC system (ThermoFisher, Milan, Italy) equipped with dual ternary pumps, an autosampler, column temperature control, and a UV/Vis detector. Separation was conducted following the UHPLC-HRMS assay conditions (Section 2.4). UV spectra were acquired over 200–600 nm, and detection of target compounds was performed at 365 nm for quercetin derivatives and 520 nm

for cyanidin derivatives, corresponding to their maximum absorbance. External standard method was employed to determine the levels of main skin compounds. Stock solutions of commercial standards Quercetin (Q) and cyanidin 3 glucoside (CyG) were prepared in MeOH at a concentration of 1 mg mL^{-1}, and stored at −20 °C. The calibration levels were prepared from the stock solutions by appropriate serial dilutions with MeOH/H_20 1:1, *v/v*, and analyzed in triplicate. The linearities of the calibration curves were evaluated in the concentration range of 3.12–75 µg mL^{-1} for Q and CyG. The regression curves were tested with the analysis of variance (ANOVA) and linear model was found appropriate over the tested concentration range (R^2 values > 0.998). The samples were analyzed after appropriate dilutions, to fall within the dynamic calibration range.

y = 0,2963x - 0,3192 calibration curve of CyG; R^2 0.999

y = 0,7659x - 0,1222 calibration curve of Q; R^2 0.998

Figure S1. Full unedited western blotting gels for figure 7 (a, b, c, d)

Full unedited gel for FIGURE 7 (a): (MFN2 and GAPDH in total cell lysates). The images shown below are the cropped membrane overlays with the MFN2 and GAPDH images detected through the iBright 1500 imager. Control, palmitate (PA) treated-, standardized bioactive ingredient (OPI-T50) treated-, and OPI-T50 + PA treated- cells samples were loaded together in the same gel, in biological triplicates.

a)

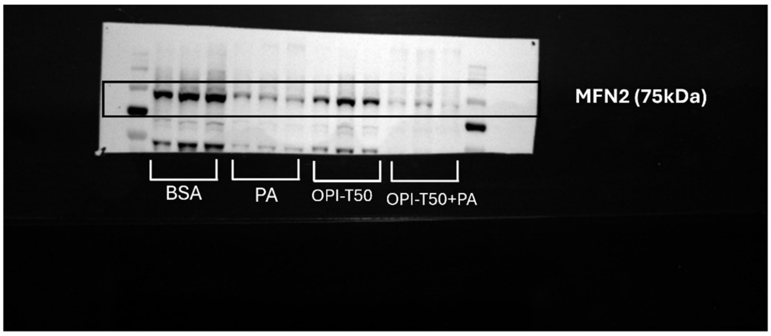

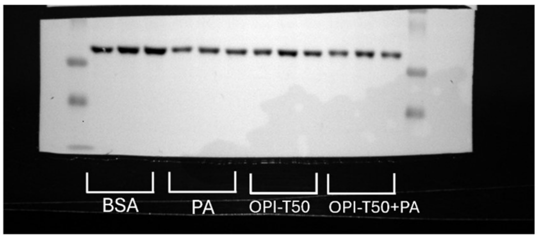

Full unedited gel for FIGURE 7 (b): (DRP1 and GAPDH in total cell lysates). The images shown below are the cropped membrane overlays with the DRP1 and GAPDH images detected through the iBright 1500 imager. Control, palmitate (PA) treated-, standardized bioactive ingredient (OPI-T50) treated-, and OPI-T50+PA treated- cells samples were loaded together in the same gel, in biological triplicates.

b)

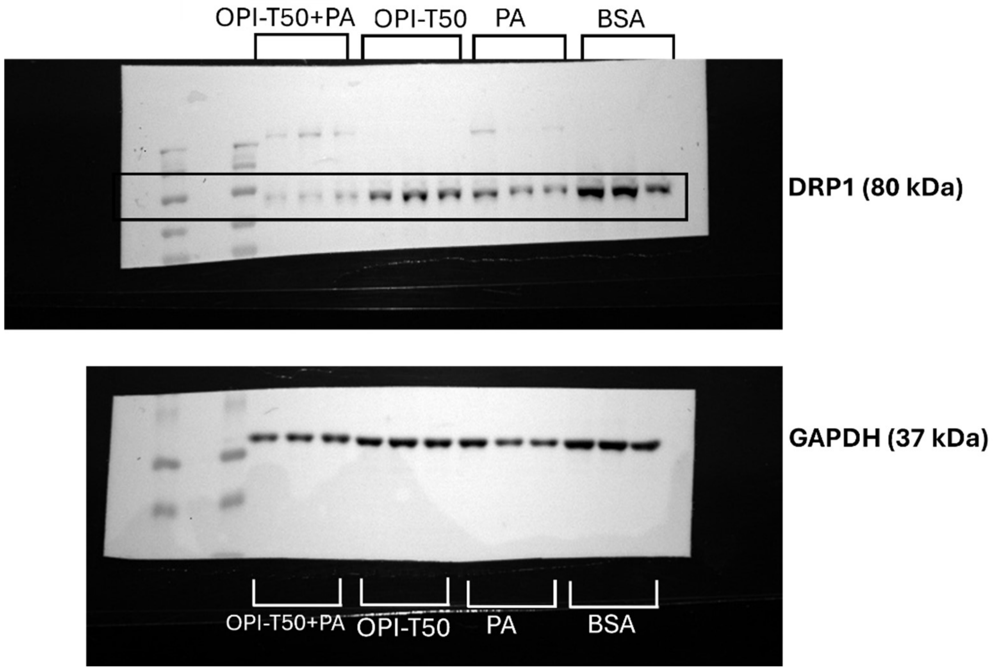

Full unedited gel for FIGURE 7 (c): (MFN2 and GAPDH in total cell lysates). The images shown below are the cropped membrane overlays with the MFN2 and GAPDH images detected through the iBright 1500 imager. Control, palmitate (PA) treated-, Montoro coppery onion peel (M50) treated-, and M50+PA treated- cells samples were loaded together in the same gel, in biological triplicates.

c)

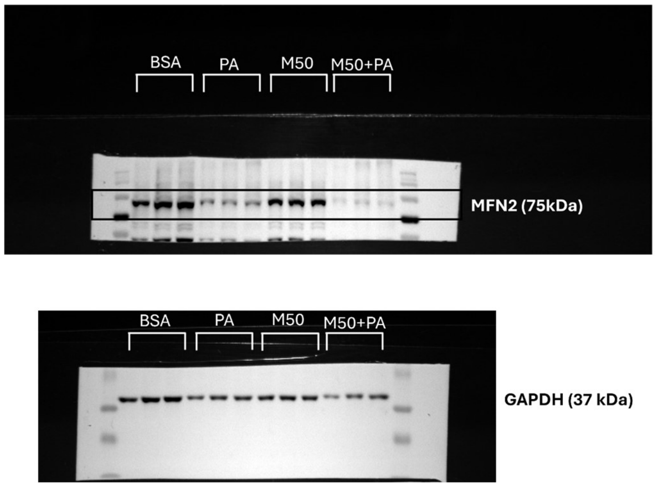

Full unedited gel for FIGURE 7 (d): (DRP1 and GAPDH in total cell lysates). The images shown below are the cropped membrane overlay with the DRP1 and GAPDH images detected through the iBright 1500 imager. Control, palmitate (PA) treated-, Montoro coppery onion peel (M50) treated-, and M50+PA treated- cells samples were loaded together in the same gel, in biological triplicates.

d)

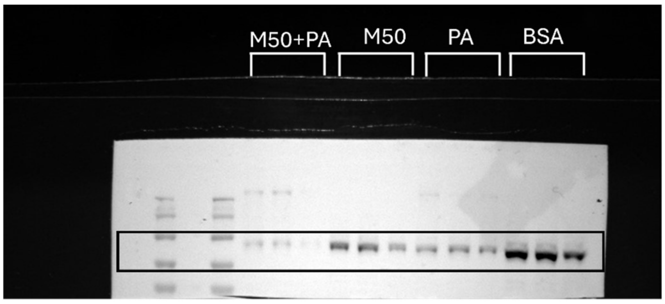

DRP1 (80 kDa)

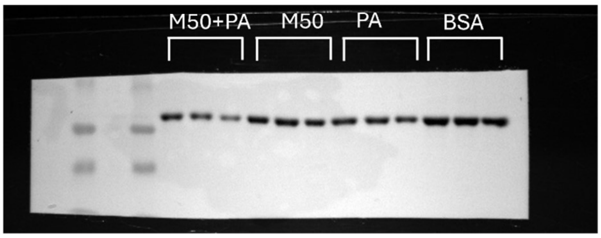

GAPDH (37 kDa)

Figure S2. Full unedited western blotting gels for and figure 8 (e, f)

The images show the full unedited gels (membrane overlays) of FIGURE 8 (e) obtained with iBright 1500 (Invitrogen – Thermofisher Scientific) Imaging System for western blotting. After the blocking phase of the western blotting, the membrane was NOT cropped, but incubated in three different times with P62, LC3 II/I and Tubulin. Therefore, the loading control tubulin was the same for both autophagy markers. Control, palmitate (PA) treated-, standardized bioactive ingredient (OPI-T50) treated-, and OPI-T50 + PA treated- cells samples were loaded together in the same gel in biological triplicates.

e)

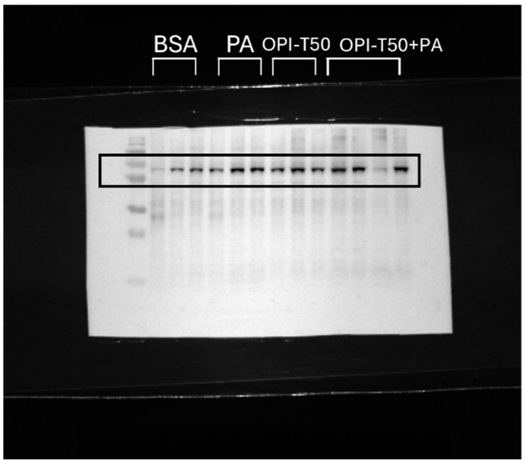

P62 (62 kDa)

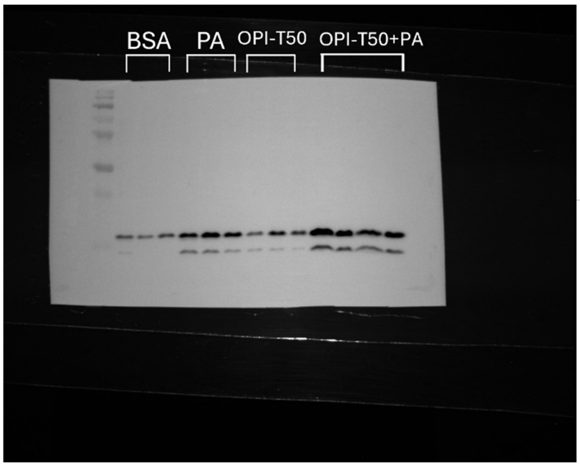

LC3 I
LC3 II (14-16 kDa)

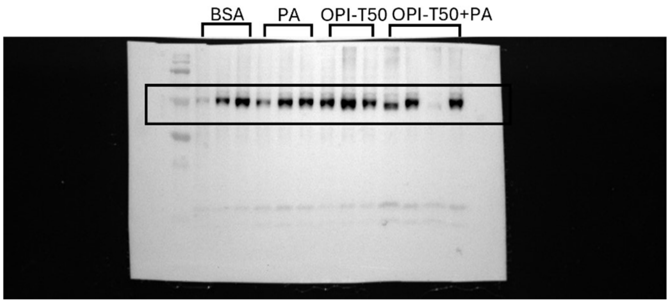

Tubulin (50 kDa)

The images show the full unedited gels (membrane overlays) of FIGURE 8(f) obtained with iBright 1500 (Invitrogen – Thermofisher Scientific) Imaging System for western blotting. After the blocking phase of the western blotting, the membrane was NOT cropped, but incubated in three different times with P62, LC3 II/I and Tubulin. Therefore, the loading control tubulin was the same for both autophagy markers. Control, palmitate (PA) treated-, Montoro coppery onion peel (M50) treated-, and M50+PA treated- cells samples were loaded together in the same gel, in biological triplicates.

f)

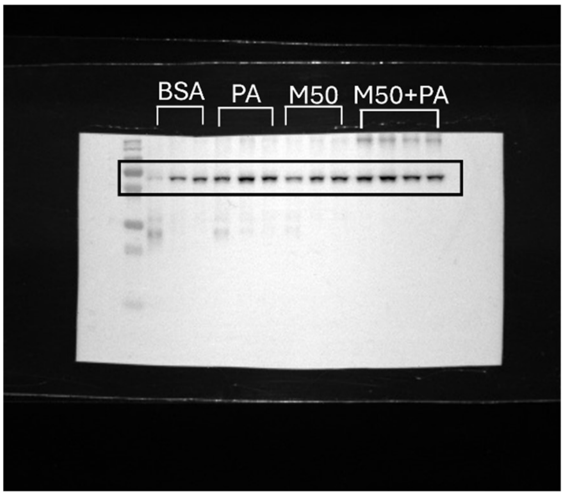

P62 (62 kDa)

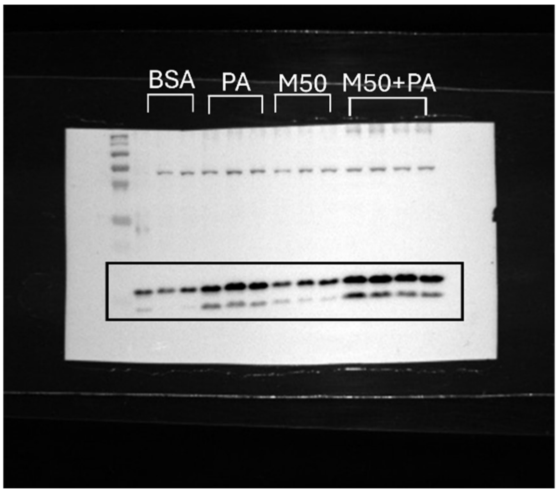

LC3 I
LC3 II (14-16 kDa)
